# Left ventricular twist and shear-angle in patients with mitral regurgitation

**DOI:** 10.1186/1532-429X-15-S1-P92

**Published:** 2013-01-30

**Authors:** Meral Reyhan, Ming Li, Himanshu Gupta, Steven G Llyod, Louis J Dell'Italia, Hyun J Kim, Thomas S Denney, Daniel Ennis

**Affiliations:** 1 Department of Radiological Sciences, University of California, Los Angeles, CA, USA; 2 Biomedical Physics Interdepartmental Program, University of California, Los Angeles, CA, USA; 3 Department of Electrical and Computer Engineering, Auburn University, Auburn, AL, USA; 4 Division of Cardiology, University of Alabama, Birmingham, AL, USA

## Background

Mitral regurgitation(MR) is a common valvular disorder that foments left ventricular(LV) dysfunction. The study of LV twist during the progression of MR is limited to studies performed with ultrasound/echo(1-3) in human subjects or animal studies(4), which suggest that LV twist decreases when MR is present. LV circumferential-longitudinal shear angle (CL-shear angle) has been proposed as a more reproducible measure of LV rotational mechanics, but has not been evaluated in patients with MR. We hypothesized that both LV twist and CL-shear angle would decrease with severity of MR.

## Methods

Normal subjects(n=54), moderate MR patients(n=29), and severe MR patients(n=54) were studied after obtaining informed consent. MRI was performed on a 1.5T scanner (Signa, GE Healthcare, Milwaukee, WI) and grid tagged LV images were collect from the base to the apex(7). LV twist and CL-shear angle measurements were derived from Fourier Analysis of STimulated echoes(FAST)(8). LV twist is defined as the difference in rotation at the apex relative to the base of the heart. Shear-angle is defined as the difference in rotation at the apex times the radius of the apex relative to the base times the radius of the base, divided by the distance between the apex and base(5, 6). Peak LV twist and peak CL-shear angle from the three groups were compared using a one-way ANOVA and Tukey's least significant difference(LSD) procedure for multiple comparisons.

## Results

Mean peak LV twist for normal subjects, moderate MR patients, and severe MR patients were: 11.5±3.2, 9.0±3.0, 8.8±2.6, respectively. Mean peak CL-shear angle for normal subjects, moderate MR patients, and severe MR patients were: 5.0±1.4, 4.7±1.6, 5.0±1.3 respectively. The one-way ANOVA of peak twist between the groups, showed differences among the groups(p<0.0001). Further investigation with LSD, showed a significant difference between the normal subjects and the moderate MR patients and between the normal subject group and the severe MR patients. However, the one-way ANOVA of peak shear-angle did not reveal any differences between the three groups(p=0.4).

## Conclusions

Peak LV twist has been shown to decrease in patients with MR compared with normal subjects, but peak CL-shear angle does not exhibit the same decrease. The pseudo-normalization of CL-shear angle in moderate and severe MR can be explained by an increase in the basal epicardial radius and a decrease in apical rotation in patients compared with normal subjects. LV twist may be a better biomarker than CL-shear angle for LV dysfunction in patients with MR.

## Funding

Funding from AHA and UCLA to MLR and NIH to DBE.

**Table 1:** 

Measurement (Peak)	Normal	Moderate MR	Severe MR
Twist [deg]	11.5±3.3	9.2±3.0	8.8±2.6
CL-shear angle [deg]	5.0±1.4	4.7±1.6	5.0±1.3
Apical Rotation [deg]	7.5±3.6	5.8±2.7	6.2±2.8
Basal Rotation [deg]	-3.9±1.3	-3.4±1.4	--2.8±1.6
Epicardial radius Apex [mm]	20.7±3.1	19.9±3.3	24.9±3.8
Epicardial radius Base [mm]	30.7±3.8	30.7±2.6	35.8±3.2
Distance between Apex and Base [cm]	5.2±0.6	4.8±0.8	4.9±0.6
Number of subjects	54	29	54
